# Evidence that circulating proteins are more promising than miRNAs for identification of patients with squamous cell carcinoma of the tongue

**DOI:** 10.18632/oncotarget.21402

**Published:** 2017-09-30

**Authors:** Linda Boldrup, Giuseppe Troiano, Xiaolian Gu, Philip Coates, Robin Fåhraeus, Torben Wilms, Lena Norberg-Spaak, Lixiao Wang, Karin Nylander

**Affiliations:** ^1^ Department of Medical Biosciences/Pathology, Umeå University, SE – 901 85 Umeå, Sweden; ^2^ Department of Clinical and Experimental Medicine, University of Foggia, 71122 Foggia, Italy; ^3^ RECAMO, Masaryk Memorial Cancer Institute, 656 53 Brno, Czech Republic; ^4^ Institut de Génétique Moléculaire, Université Paris 7, Hôpital St. Louis, 75010 Paris, France; ^5^ Department of Clinical Sciences/ENT, Umeå University, SE – 901 85 Umeå, Sweden

**Keywords:** miRNA, circulating markers, NT-3, miR-150, squamous cell carcinoma of the tongue

## Abstract

Despite intense research, squamous cell carcinoma of the tongue remains a devastating disease with a five-year survival of around 60%. Late detection and recurrence are the main causes for poor survival. The identification of circulating factors for early diagnosis and/or prognosis of cancer is a rapidly evolving field of interest, with the hope of finding stable and reliable markers of clinical significance. The aim of this study was to evaluate circulating miRNAs and proteins as potential factors for distinguishing patients with tongue squamous cell carcinoma from healthy controls. Array-based profiling of 372 miRNAs in plasma samples showed broad variations between different patients and did not show any evidence for their use in diagnosis of tongue cancer. Although one miRNA, miR-150, was significantly down-regulated in plasma from patients compared to controls. Surprisingly, the corresponding tumor tissue showed an up-regulation of miR-150. Among circulating proteins, 23 were identified as potential markers of squamous cell carcinoma of the tongue. These findings imply that circulating proteins are a more promising source of biomarkers for tongue squamous cell carcinomas than circulating miRNAs. The data also highlight that circulating markers are not always directly associated with tumor cell properties.

## INTRODUCTION

Squamous cell carcinoma of the tongue (SCCT) is a devastating disease with a 5-year survival of around 60% [1] which has not improved over the last decades [2]. One major problem is that around 60% of patients develop a local relapse [3], the main cause (24%) of death for SCCT [4]. It is therefore of great importance to find markers for prediction and early detection of relapses. However, it has been challenging to identify biomarkers of clinical significance for SCCT due to the heterogeneous nature of this tumor type [5].

Circulating miRNAs were discovered in 2008 [6], 15 years after the discovery of miRNA in tissue [7]. While the majority of miRNAs exist intracellularly, some enter the circulation. miRNAs have been isolated from blood, urine, saliva, pancreatic juice and breast milk [8]. How miRNAs enter these body fluids is poorly understood [9, 10], but is suggested to be via passive leakage from cells due to necrosis, apoptosis or inflammation, through active release by complex formation with lipoproteins (HDL) or RNA binding proteins (AGO2), or by packaging in exosomes or microvesicles before being released [10–12], or a combination of these events.

Regardless of how they are released, cell-free miRNAs are stable, even when subjected to extreme conditions such as high temperature, wide variation in pH or multiple freeze-thaw cycles [13, 14]. Although there are inconsistencies between studies, some have suggested that miRNA profiles in plasma can differentiate patients with oral squamous cell carcinoma (OSCC) from healthy individuals (reviewed in [15]).

Circulating factors are potentially ideal biomarkers for simple, rapid and inexpensive screening of patients for cancer diagnosis and monitoring, such as the use of prostate-specific antigen for prostate [16] or CA125 for ovarian cancer [17]. In both cases, these proteins can be measured easily to provide diagnostic information and to monitor therapeutic response and tumor relapse, but suffer from problems with specificity and/or sensitivity. Circulating proteins have also been evaluated in patients with squamous cell carcinoma of the head and neck (SCCHN) [18–20], although not as extensively as miRNAs. Studies using panels of cytokines and growth factors have found promising markers for early detection of SCCHN [18, 20]. For example, macrophage inflammatory protein 1b (MIP 1b), interleukin 13 (IL13), metalloproteinase 3 (MMP3), epidermal growth factor (EGF) and vascular cell adhesion molecule (VCAM) are down-regulated in plasma from SCCHN patients compared to controls. Furthermore, both squamous cell carcinoma antigen (SCC-Ag) and neurite growth-promoting factor 2 (Midkine) have been suggested as independent prognostic factors for patients with SCCHN [21, 22].

Unlike detection of circulating nucleic acids that can be amplified prior to detection using array technologies, two potential problems with serum protein biomarkers are their low concentrations due to dilution in the circulation and the limited ability to assay multiple proteins from a small sample volume. Proximity extension assay (PEA) is a highly sensitive method based on pairs of antibodies linked to oligonucleotides with partial complementariness for each other (PEA probes). When the two different antibodies bind to their target protein, probes are brought into proximity and the two oligonucleotides accordingly bind. The new sequence thus achieved can be extended, amplified and measured by quantitative real-time PCR [23, 24], where the number of PCR templates is proportional to the initial concentration of antigen in the sample. In addition to high sensitivity, specificity is also improved due to the requirement for simultaneous binding of two different antibodies to epitopes on the same antigen. However, the technique is dependent on cross reactivity and sensitivity of the antibodies used. Recent technological advances have enabled simultaneous measurement of multiple proteins, and a 96-plex PEA-based immunoassay has made it possible to analyze 92 known or putative biomarkers simultaneously [25].

In this study we have compared two different sensitive technologies for the detection of miRNAs or proteins in plasma as potential diagnostic markers for squamous cell carcinoma of the tongue.

## RESULTS

### miR-150 down-regulation in plasma and up-regulation in tissue from patients with SCCT compared to healthy controls

A panel of circulating miRNAs was analyzed in plasma from healthy individuals (13 controls) and patients with SCCT (13 patients). Of the 372 miRNAs analyzed, 152 were detected in all samples. None of the samples showed signs of hemolysis, evaluated by analyzing the ratio between miR-451 and miR-23a-3p. All samples had values below 7.0, where a ratio above 7.0 is considered as risk of being affected by hemolysis.

**Figure 1:**
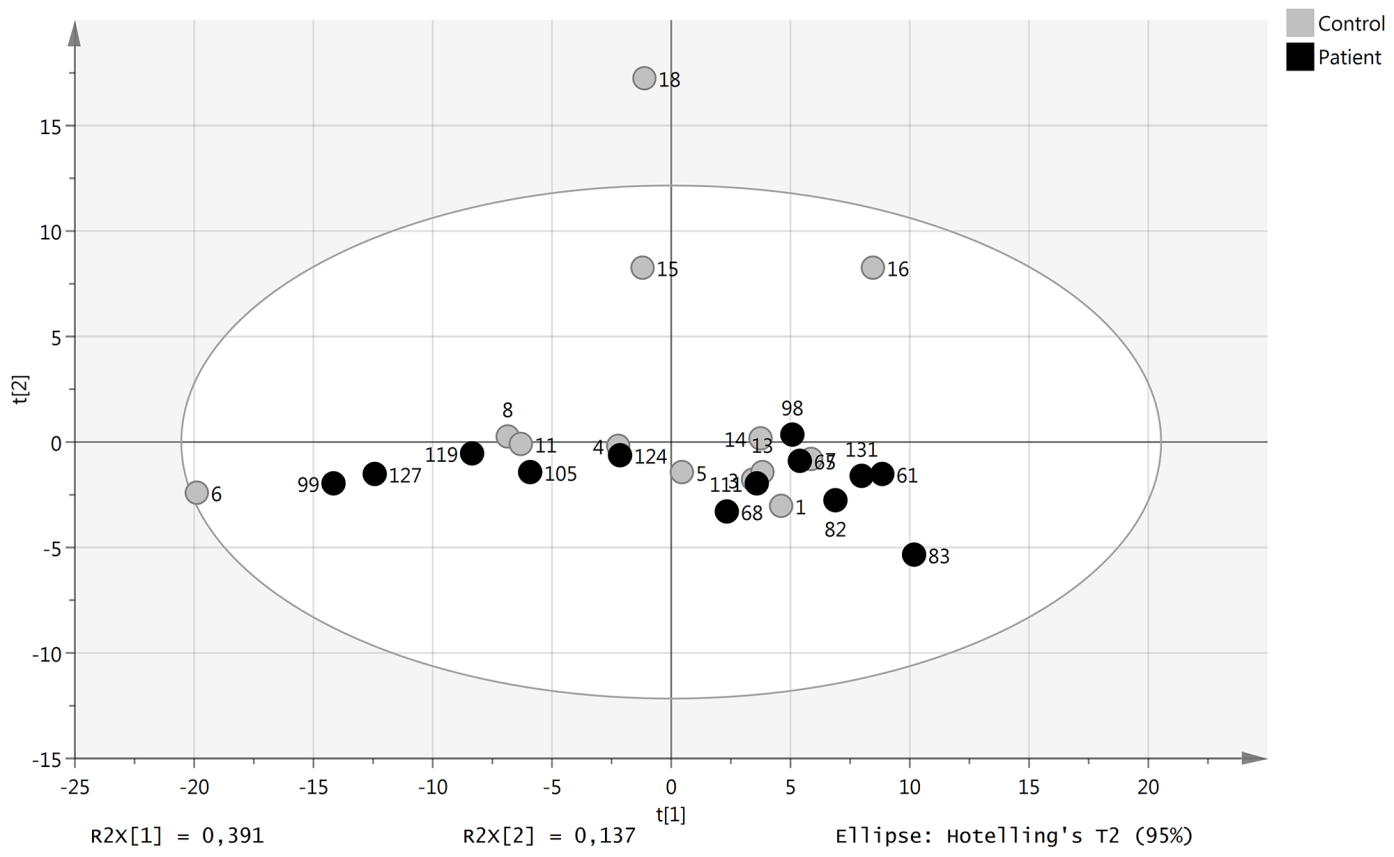
miRNA levels in patients with tongue tumor compared with healthy controls Score plot (t1/t2) from PCA modelling based on 152 miRNAs detected in all samples. Each dot represent one patient indicated by the patient ID.

**Figure 2:**
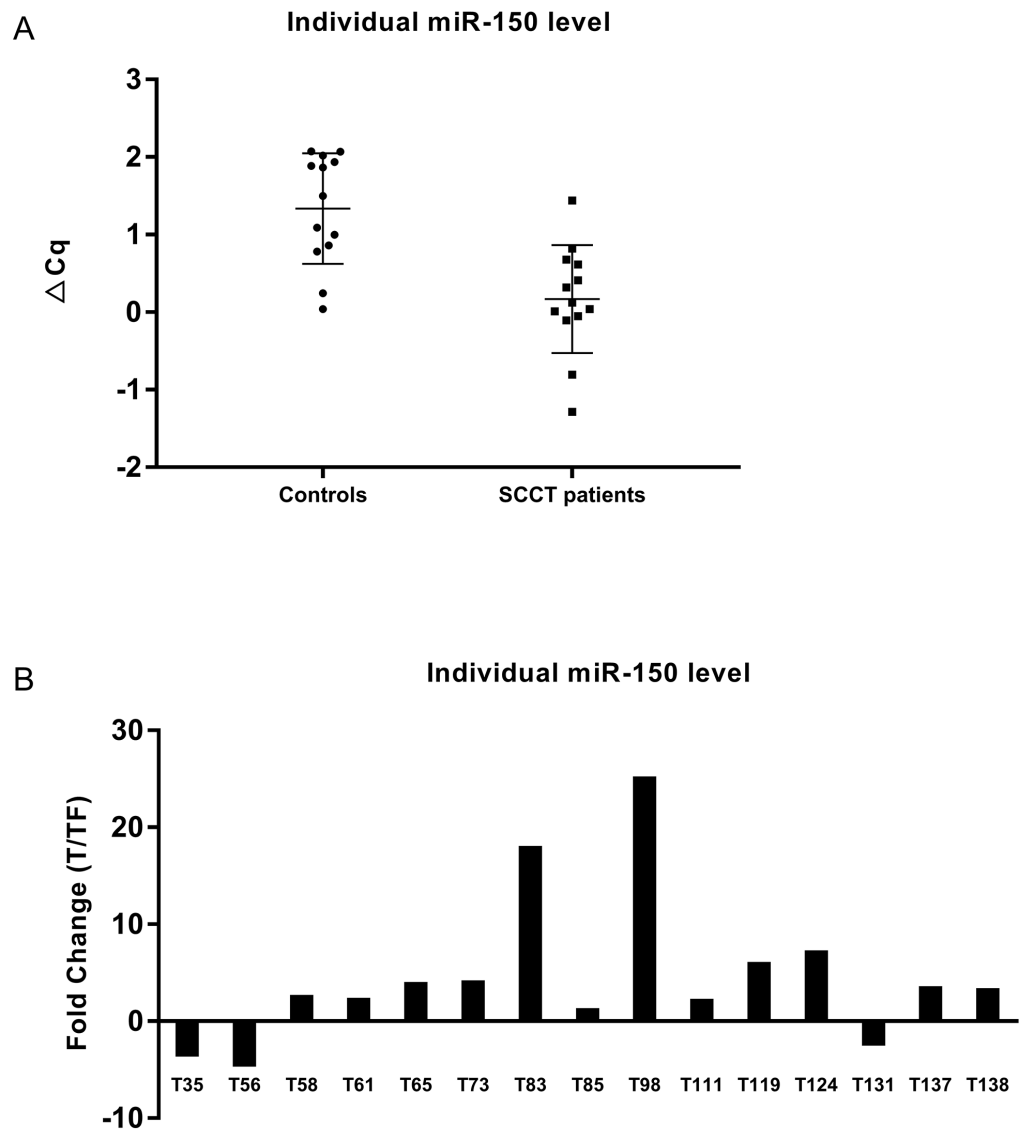
miR-150 levels in plasma and tissue **(A)** Individual miR-150 levels in plasma from controls and tumors based on Cq values from the miRNA-panel. Normalized Cq = global mean Cq – assay Cq (sample). A higher value thus indicate that miR-150 is more abundant in that particular sample. **(B)** Fold change in miR-150 level in tongue tumor compared to tumor free tongue tissue from the same patient.

Principal component analysis (PCA) showed that the levels of these miRNAs did not discriminate healthy individuals from SCCT patients (Figure 1). Statistical evaluation of individual miRNA levels showed miR-150 to be significantly decreased in patients with SCCT compared to controls, Benjamini-Hochberg FDR adjusted p-value 0.007 (Figure 2A). Single qRT-PCR assay was then applied to validate miR-150 results using a combination of three miRNAs for normalization (miR-93, miR-222 and miR-30e). The normalized Cq value from single qRT-PCR assays of plasma samples showed high consensus with the normalized Cq value from the panel (p < 0.001, Rho = 0.913).

miR-150 was further analyzed in tissue samples using paired tumor and tumor free samples from 15 SCCT patients; 8 of the patients included in the plasma analysis and an additional 7 patients. In contrast to the decreased plasma levels in tumor patients, there was significant up-regulation (p-value 0.02) of miR-150 in tumor tissue compared to tumor free tissue adjacent to the tumor (Figure 2B). There was no correlation between plasma and tissue miR-150 in the 8 patients for whom both sets of data were available. One well known target for miR-150 is the *MYB* proto-oncogene. RNA expression of *MYB* was down-regulated in all SCCT samples compared to tumor free controls adjacent to tumor [26]. However, there was no direct correlation between miR-150 and *MYB* levels.

### Using proximity extension assay 23 proteins separated SCCT patients from healthy individuals

**Figure 3:**
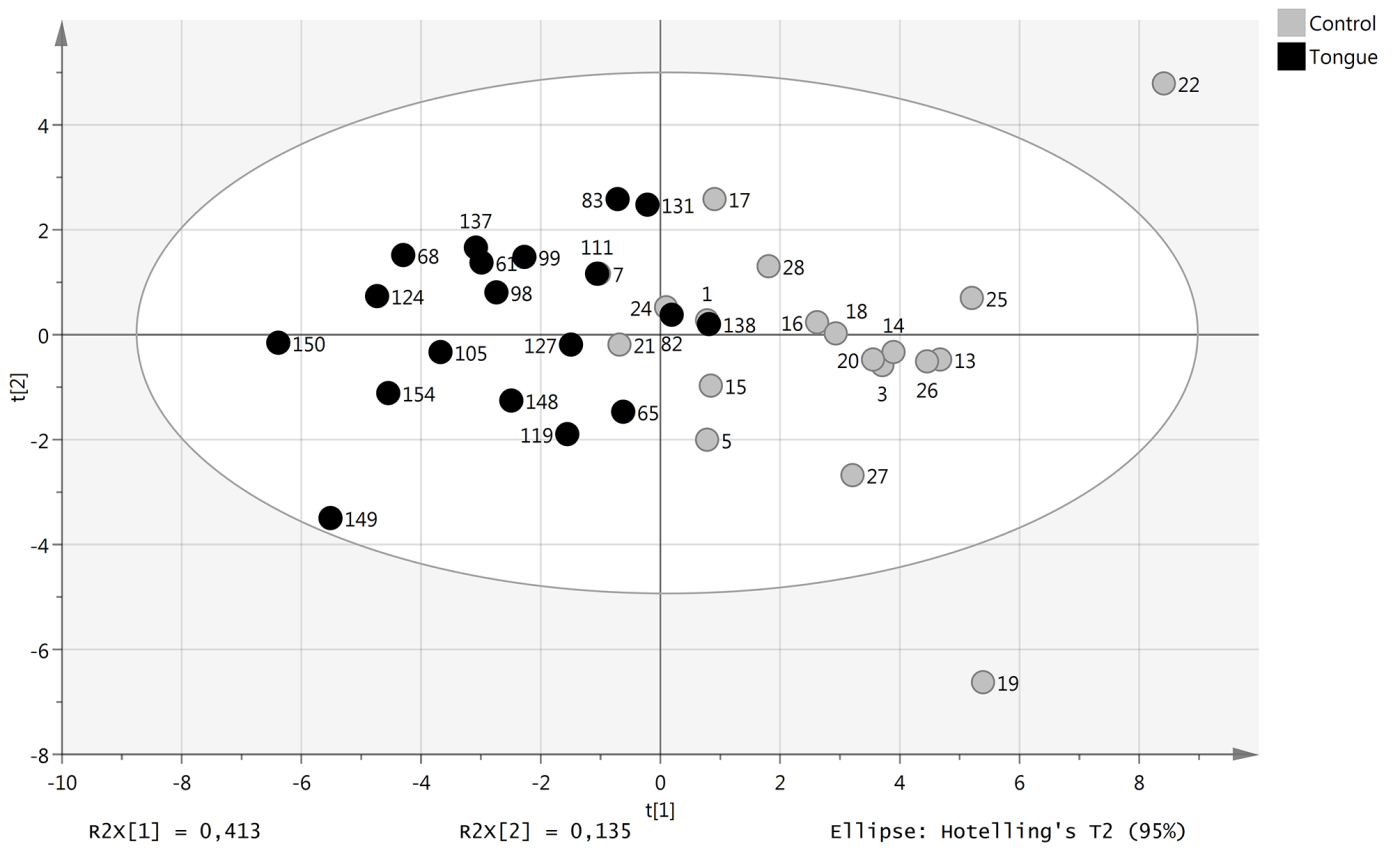
Levels of plasma proteins with tongue tumor compared with healthy controls Score plot (t1/t2) from PCA modelling based on 23 plasma proteins with significantly different levels in patient and control samples.

**Figure 4:**
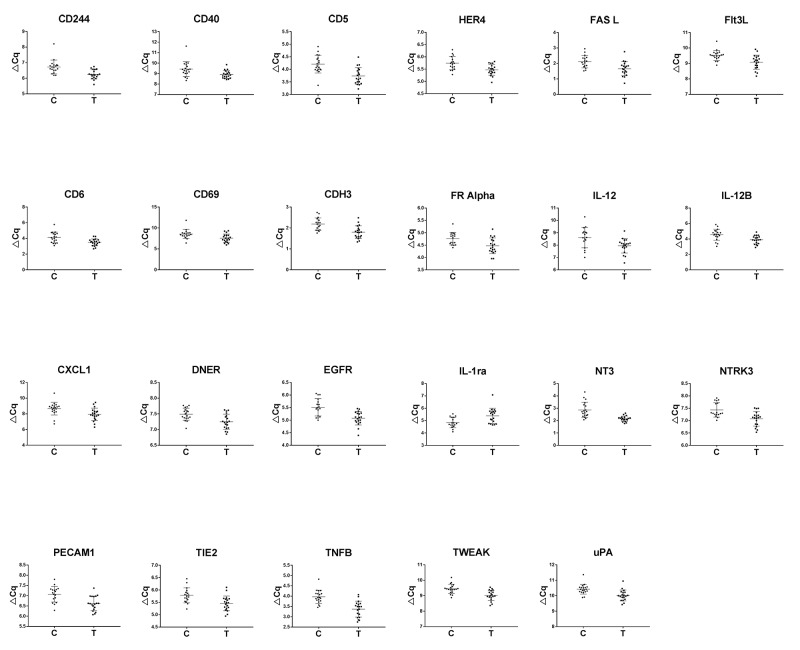
Differentially expressed proteins Scatter plots of significantly differentially expressed proteins in plasma from patients with squamous cell carcinoma of the tongue compared to healthy controls.

A total of 146 proteins involved in oncogenesis and inflammation were analyzed by proximity extension assay of plasma from 19 SCCT patients (the same 13 as included in the miRNA analysis and an additional 6 patients) and 19 healthy controls (9 that were included in the miRNA analysis and 10 additional controls). Using a cut-off of p < 0.05, 23 markers were differentially expressed between SCCT patients and healthy individuals. Principal component analysis of these 23 proteins separated SCCT patients and healthy individuals from each other, although not completely (Figure 3). All but one of these proteins showed down-regulation in SCCT patients compared to healthy controls, the exception being IL1-ra, and there was a large overlap in protein levels between the groups for all proteins (Figure 4). Calculating a receiver operating curve (ROC) showed the AUC for individual proteins to range from 0.676 to 0.903, indicating fair to good discrimination between those with and without disease (Table 1). The protein with highest AUC value was NT-3 (neurotrophin-3; NTF3), which is also the only candidate with both high sensitivity and high specificity.

**Table 1:** Protein levels in circulation and mRNA expression in tissue

	Protein levels in circulation		mRNA expression in tissue ^1^		AUC Circulating proteins		
						Confidence interval	
	p-value	SCCT vs C	P-value	SCCT vs C	AUC	Lower Bound	Upper Bound
NT-3	0,002	Down*	1,11E-02	Down*	0,903	0,805	1,000
TNFB	0,001	Down*	6,64E-05	Up*	0,889	0,786	0,993
CD5	0,008	Down*	4,04E-04	Up*	0,831	0,694	0,968
uPA	0,014	Down*	1,21E-10	Up*	0,825	0,686	0,965
IL-1ra	0,031	Up*	3,77E-05	Down*	0,806	0,670	0,942
Flt3L	0,018	Down*	1,04E-02	Up*	0,803	0,656	0,950
DNER	0,031	Down*	1,08E-05	Down*	0,781	0,631	0,931
CXCL1	0,047	Down*	2,31E-06	Up*	0,770	0,607	0,933
CD6	0,018	Down*	2,02E-07	Up*	0,767	0,615	0,919
CD40	0,047	Down*	4,69E-07	Up*	0,765	0,605	0,924
CDH3	0,014	Down*	1,80E-11	Up*	0,737	0,570	0,904
PECAM-1	0,014	Down*	1,21E-02	Up*	0,731	0,560	0,902
TIE2	0,031	Down*	2,22E-02	Down*	0,712	0,538	0,886
FasL	0,031	Down*	7,72E-03	Up*	0,709	0,538	0,881
FR-alpha	0,047	Down*	3,55E-02	Up*	0,693	0,519	0,867
CD69	0,044	Down*	4,69E-07	Up*	0,676	0,495	0,856
CD244	0,012	Down*	5,33E-01	Down	0,845	0,723	0,967
TWEAK	0,010	Down*	6,71E-01	Down	0,834	0,705	0,962
IL-12B	0,026	Down*	4,23E-01	Down	0,795	0,644	0,946
EGFR	0,008	Down*	2,58E-01	Up	0,756	0,593	0,920
NTRK3	0,016	Down*	2,58E-01	Down	0,731	0,562	0,900
ErbB4/HER4	0,031	Down*	6,71E-01	Down	0,690	0,515	0,864
IL-12	0,047	Down*	6,47E-01	Up	0,676	0,487	0,865

^*^ Significant differential expression

^1^Data from Boldrup et al. 2017 ([26])

### *DNER, NT3* and *TIE2* mRNA are also down-regulated in tumor tissue

For the 23 differentially expressed proteins, corresponding gene expression was available in a recently published study of RNA profiling in nine of the patients [26]. Sixteen mRNAs showed significantly different expression when comparing tumor tissue to control (Table 1; upper part), whilst mRNA levels for 7 of the proteins was not significantly different between control and tumor tissues (Table 1, lower part). Consistency in the direction of change for circulating protein and tissue mRNA levels were identified in only three of the 16 differentially expressed genes (*DNER*, *NT3* and *TIE2*), where protein levels were lower in plasma from patients than controls and mRNA levels were lower in tumor tissue compared to tumor-free tissue. The remaining thirteen genes that were significantly dysregulated in tumor tissue showed the opposite direction when comparing mRNA to corresponding circulating protein.

## DISCUSSION

The use of circulating markers for diagnosis, prognosis and disease monitoring has been employed for many years for specific cancer types. However, such markers suffer from a lack of specificity and/or sensitivity, and are only applicable to a limited number of cancer types. With the advent of new technologies, many reports are appearing for the identification of new disease markers. Here, we evaluated the potential value of circulating miRNAs and proteins in distinguishing patients with squamous cell carcinoma of the mobile tongue (SCCT) from healthy controls. In contrast to previous studies on plasma suggesting that, for example, miR-21, miR-31, miR-184 and miR-186-5p are altered in patients with SCCHN compared to controls [27–30], we found only miR-150 to be significantly different in plasma from SCCT patients, a specific subgroup of SCCHN.

The discrepancies seen between studies of miRNA is one of many challenges when evaluating circulating miRNAs as potential biomarkers (reviewed in [31]). Factors known to affect results are choice of method for normalization and the use of different patient cohorts, making comparisons between studies difficult. In contrast to many of the previous studies that included tumors originating from the whole head and neck area and the majority being N+ [27–29], we only included patients with SCCT, with the majority (11/13) being N0. The importance of taking sub-site into consideration in analysis of SCCHN has previously been shown by us and others [32–34].

Results showed high variation in miRNA levels within controls, even though samples had been collected in the same way and analyzed using the same method on the same day and by the same person. This again could reflect the dynamic expression of miRNA and the importance of global protocols [35]. Even though there was a significant down-regulation of miR-150 in SCCT patients in group wise comparison, only two patients had a lower value than the mean minus two S.D. of the controls. In a previous study of colorectal cancer, lower plasma levels of miR-150 were seen in patients with advanced cancer compared to patients with polyps and adenomas. Also here the variance within the groups was large, particularly within the control group [36].

Various mechanisms of how tumors release miRNAs into the circulation have been suggested [37] making it difficult to judge if miRNA levels in plasma really reflect miRNA expression in tumor tissue. It has been proposed that part of the circulating miRNAs originate from blood cells and that the miRNA content therefore reflects the blood count. miR-150 is strongly correlated with lymphocyte count [38] and specifically expressed by mature lymphocytes [39], but can also be expressed at a lower level by keratinocytes [40]. One of the top targets for miR-150 is *MYB*, a transcription factor essential during lymphocyte development, especially B cell development [41–43]. In accordance with previous findings, *MYB* mRNA was down-regulated in all our SCCT samples compared to tumor free controls adjacent to tumor. However, no direct correlation between miR-150 and *MYB* was seen, indicating that other factors are also important in regulation of *MYB*.

In our analysis of circulating proteins involved in tumor development and inflammatory processes, 23 were differentially expressed and could aid in distinguishing SCCT patients from controls. A previous report including patients with tumors in the oral cavity, pharynx and larynx using an equivalent method measuring 60 cytokines, growth factors and tumor antigens from SCCHN patients [20] presented a panel of 25 proteins as a promising new approach for early detection of head and neck cancer. Seven of the proteins included in that panel were also included in our panel (CEA, ErbB2, EGFR, IL-1Ra, IL-8, HGF and IL-6) but only two showed significant alteration (EGFR and IL-1Ra) in patients with SCCT.

In our material, 16 of the 23 proteins analyzed showed significant dysregulation both at RNA level in tissue and protein level in plasma. However, only three genes/proteins, DNER, NT-3 and TIE2 showed the same direction of down-regulation in both plasma and tumor tissue. Using area under the curve (AUC) analysis, neurotrophin-3 (NT-3, or NTF3) showed the best discrimination between controls and SCCT patients. Apart from being connected to somatosensory innervation in the mouse tongue [44], NT-3 also plays a role in breast cancer [45], lung cancer [46] and adenoid cystic cancer [47]. Based on its role in metastatic growth, tumor behavior and invasion in these tumor types, its role in SCCT will be worth further elucidation.

As circulating proteins show promising results as potential markers for SCCT, future studies will aim at clarifying their role in a bigger group of stage I tumors only where such markers could be of value in early diagnosis.

In summary we have shown the potential of circulating proteins as markers of squamous cell carcinoma of the mobile tongue. In particular, NT-3 with known roles in other cancer types requires further investigation in SCCT. For circulating miRNAs, our results were less promising, showing wide inter-individual variations and even for the best performing miRNA, miR-150, only two patients had levels outside the area of the controls. With the detection tools available today, circulating miRNAs are unlikely to be valuable in the diagnosis/monitoring of SCCT. However an extended panel of circulating proteins and the PEA assay is a promising approach for detection of clinically useful markers in the future.

## MATERIALS AND METHODS

### Patient material

The study was approved by the local Ethical committee (Dnr 08-003M). After informed consent from all participants diagnosed with primary SCCT, blood or/and tissue biopsies were collected before treatment of the SCCT. Whole blood was collected from a total of 19 patients and 23 healthy volunteers, mean age 58 and 50 years respectively. Tumor biopsies (T) and biopsies from corresponding clinically normal tumor free tissue (TF) adjacent to the tumor was collected from 15 patients, with a mean age of 59 years. Detailed patients information is shown in Table 2. All biopsies were collected from mobile tongue. Samples were snap-frozen in liquid nitrogen and stored at -80°C until miRNA was extracted.

**Table 2:** Characteristics and clinical parameters of tongue squamous cell carcinoma patients and controls

							Circulating miRNA			
Patient ID	Gender	Age	TNM	Survival	Recurrence	Panel	Verification	miRNA tissue	Protein plasma	RNA tissue
35	F	24	T2N0M0	Dead	Yes			X		X*
56	F	41	T2N2bM0	Dead	Yes			X		X*
58	M	62	T1N0M0	Alive	No			X		X*
61	M	70	T4aN0M0	Alive	No	X	X	X	X	X*
65	F	81	T2N0M0	Alive	No	X	X	X	X	X*
68	M	62	T2N0M0	Dead	Yes	X	X		X	X*
73	M	81	T4aN0M0	Dead	No			X		X*
82	F	19	T4aN0M0	Dead	Yes	X	X		X	X*
83	F	64	T1N0M0	Alive	No	X	X	X	X	
85	F	88	T2N0M0	Dead	Yes			X		X*
98	M	31	T2N0M0	Alive	No	X	X	X	X	
99	M	65	T4aN2cM0	Alive	No	X	X		X	
105	M	64	T1N0M0	Alive	No	X			X	
111	F	31	T1N0M0	Alive	No	X	X	X	X	
119	M	67	T2N0M0	Alive	No	X	X	X	X	
124	M	54	T4N2bM0	Dead	Never TF	X	X	X	X	
127	M	28	T1N0M0	Alive	No	X	X		X	
131	F	74	T2N0M0	Alive	No	X	X	X	X	
137 ¤	F	71	T2N0M0	Alive	No			X	X	
138 ¤	M	50	T2N1M0	Alive	No			X	X	
148 ¤	M	81	T1N0M0	Alive	No				X	
149 ¤	F	69	T1N0M0	Alive	No				X	
150 ¤	M	79	T3N0M0	Alive	No				X	
154 ¤	F	42	T1N1M0	Alive	No				X	

^*^Data published in Boldrup et al, 2017 ([26])

^¤^ Follow up time less than 2 years

**Table:** 

Controls			
	n	Mean age	Age range
**Circulating miRNAs**			
Female	7	46,4	21- 82
Male	6	44,7	23- 59
**miRNA verification plasma**			
Female	7	46,4	21- 82
Male	5	43,6	23- 59
**Circulating proteins**			
Female	11	55,2	39- 82
Male	8	56,8	38- 83
RNA tissue*			
Female	9	40,3	25- 59
Male	5	39,2	27- 57

^*^Data published in Boldrup et al, 2017 ([26])

### Collection and storage of blood plasma

Three milliliters of peripheral blood was collected into vacutainers containing EDTA using standardized venipuncture procedures. Handling and processing was the same for all samples. Tubes were left standing for at least 30 minutes at room temperature after collection, centrifuged at 1300 g for 10 min at room temperature and the top layer, the plasma, was immediately aliquoted and stored at -80 °C until further use.

### miRNA extraction from plasma and tissue

Total RNA including miRNA was extracted from frozen plasma samples using miRCURY RNA Isolation kit –Biofluids (Exiqon, Vedbaek, Denmark). A standard protocol for RNA isolation was used according to the manufacturer, with 250 μl of plasma as starting material. After centrifugation to remove debris, 200 μl supernatant was extracted and RNA eluted in 50 μl RNase free water. AllPrep DNA/RNA/miRNA Universal Kit (Qiagen, Hilden, Germany) was used for miRNA extraction from tumor and tumor free tissue. The fresh frozen biopsies (less than 20 mg) were homogenized in 600 μl Buffer RLT Plus containing β-mercaptoethanol using a Precellys Tissue homogenizer (Bertin Technologies, Artigus Pres Boreaux, France). After DNase treatment and washing, total RNA including miRNA was eluted twice in 30 μl and the eluates pooled. Purified RNA was stored at -80 °C until cDNA preparation. Quantity and purity of RNA was measured using a NanoDrop ND-1000 spectrophotometer (Thermo Scientific, Wilmington, DE, USA).

### miRNA profiling in plasma

Twenty microliters of RNA extracted from plasma from 13 patients and 13 controls was sent on dry ice to Exiqon Services (Vedbæk Denmark) for miRNA analysis with real-time PCR panel. The panel used was miRCURY LNA Universal RT microRNA PCR Human panel I. Briefly 10 μl RNA was reversed transcribed in 50 μl using the miRCURY LNA Universal RT microRNA PCR, polyadenylation and cDNA synthesis kit (Exiqon, Vedbæk, Denmark). cDNA was diluted 50x and assayed in 10 μl PCR reactions according to the kit protocol. Amplification was performed in a LightCycler® 480 Real-time PCR system (Roche) in 382 well plates, and amplification curves analyzed using Roche LC software, both for determination of Cq (by the 2^nd^ derivative method) and for melting curve analysis. All samples went through a quality control of RNA isolation and cDNA synthesis using Exiqon´s RNA spike-in kit.

Data analysis: the amplification efficiency was calculated using algorithms to the LinReg software. Using NormFinder (http://moma.dk/normfinder-software), the best normalizer was the average of assays detected in all samples (average-assay Cq). All data were therefore normalized by the formula; normalized Cq = global mean Cq – assay Cq (sample).

### miRNA validation with qRT-PCR in plasma and tissue samples

For validation of findings from the panel in plasma, miR-150 and three miRNAs used for normalization, miR-30e, miR-93 and miR-222, were quantified by individual qRT-PCR assays. miR-30e, miR-93 and miR-222 were selected by Normfinder and recommendations from Exiqon (Biofluids Guidelines, http://www.exiqon.com/ls/Documents/Scientific/microRNA-serum-plasma-guidelines.pdf). All samples but one control and one SCCT were subjected to the validation.

RNU6 and SNORD 44 were used for normalization of miR-150 tissue data, obtained from 8 of the 13 SCCT samples included in the plasma analysis plus an additional 7 samples. All primers were from Exiqon. cDNA preparation and qRT-PCR analyses used the protocol for individual assays in miRCURT LNA Universal RT microRNA PCR kit from Exiqon. Two microliters of extracted RNA from plasma were used for each 10 μl cDNA reaction as recommended by miRCURY RNA Isolation kit – Biofluids. For tissue samples, 10 ng of total RNA was used in each cDNA reaction.

Real time RT-PCR was performed using an IQ5 multicolor real-time PCR detection system with IQ SYBR Green Supermix (Bio-Rad Laboratories, Hercules, CA, USA). For each assay, 4 μl cDNA, diluted 40X, was used in 10 μl reactions. Cycling conditions were enzyme activation at 95°C for 10 min followed by 45 cycles of denaturation at 95°C for 10 sec and annealing/extension at 60°C for 60 sec.

### Proximity extension assay

Plasma from 19 controls (9 of the samples included in the miRNA analysis and an additional 10) and 19 SCCT patients (12 of the 13 analyzed for miRNA in plasma and an additional 7 patients with SCCT) were sent to Clinical biomarkers facility, Science for Life Laboratory (Uppsala, Sweden) for analysis with proximity extension assay. One μl EDTA-plasma was used in each of two different panels comprising 92 and 71 proteins respectively, Proseek multiplex Oncology I, v2 and Proseek multiplex Inflammation I (Olink Bioscience, Uppsala, Sweden).

### Statistical analysis

For multivariate data analysis we used Simca –P 14 (MKS Data analytics Solutions, Umea, Sweden). Multiple Experiment Viewer (MeV) (http://www.tm4.org/mev.html) with the LIMMA package was used to identify significantly differentially expressed miRNAs from the panel and also circulating proteins from the protein panel. Spearman correlation was used for statistical correlations between miRNA panel results and results from single assays and for correlations between plasma and tissue miRNA. Wilcoxon test was used for evaluation of tissue expression of miR-150 in paired tissue samples. The predictive analytical software SPSS was used for the above statistical analysis and receiver operating characteristics (ROC) and area under the curve (AUC) analysis to test how well individual factors separate the groups. All graphs were prepared in Graphpad Prism 7 (Graphpad prism software).

## Abbreviations

AUC: Area under the curve
C: Control
DNER: Delta/Notch like EGF repeat containing
EGF: epidermal growth factor
IL13: interleukin 13
miRNA: microRNA
Midkine: neurite growth-promoting factor 2
MIP 1b: macrophage inflammatory protein 1b
MMP3: metalloproteinase 3
NT-3: Neurotrophin-3
OSCC: Oral squamous cell carcinoma
PCA: Principal component analysis
PEA: Proximity extension assay
ROC: receiver operating curve
SCC-Ag: squamous cell carcinoma antigen
SCCHN: Squamous cell carcinoma of the head and neck
T: Tumor
TF: Tumor free
TIE2: TEK Receptor tyrosine kinase
SCCT: Squamous cell carcinoma of the tongue
VCAM: vascular cell adhesion molecule

## References

[1] Siegel RL, Miller KD, Jemal A (2015). Cancer statistics, 2015. CA Cancer J Clin.

[2] Lam L, Logan RM, Luke C, Rees GL (2007). Retrospective study of survival and treatment pattern in a cohort of patients with oral and oropharyngeal tongue cancers from 1987 to 2004. Oral Oncol.

[3] Agra IM, Carvalho AL, Pinto CA, Martins EP, Filho JG, Soares FA, Kowalski LP (2008). Biological markers and prognosis in recurrent oral cancer after salvage surgery. Arch Otolaryngol Head Neck Surg.

[4] Carvalho AL, Magrin J, Kowalski LP (2003). Sites of recurrence in oral and oropharyngeal cancers according to the treatment approach. Oral Dis.

[5] Rothenberg SM, Ellisen LW (2012). The molecular pathogenesis of head and neck squamous cell carcinoma. J Clin Invest.

[6] Mitchell PS, Parkin RK, Kroh EM, Fritz BR, Wyman SK, Pogosova-Agadjanyan EL, Peterson A, Noteboom J, O’Briant KC, Allen A, Lin DW, Urban N, Drescher CW (2008). Circulating microRNAs as stable blood-based markers for cancer detection. Proc Natl Acad Sci USA.

[7] Lee RC, Feinbaum RL, Ambros V (1993). The C. elegans heterochronic gene lin-4 encodes small RNAs with antisense complementarity to lin-14. Cell.

[8] Weber JA, Baxter DH, Zhang S, Huang DY, Huang KH, Lee MJ, Galas DJ, Wang K (2010). The microRNA spectrum in 12 body fluids. Clin Chem.

[9] Chen M, Calin GA, Meng QH (2014). Circulating microRNAs as Promising Tumor Biomarkers. Adv Clin Chem.

[10] Cheng G (2015). Circulating miRNAs: roles in cancer diagnosis, prognosis and therapy. Adv Drug Deliv Rev.

[11] Jarry J, Schadendorf D, Greenwood C, Spatz A, van Kempen LC (2014). The validity of circulating microRNAs in oncology: five years of challenges and contradictions. Mol Oncol.

[12] Koberle V, Pleli T, Schmithals C, Augusto Alonso E, Haupenthal J, Bonig H, Peveling-Oberhag J, Biondi RM, Zeuzem S, Kronenberger B, Waidmann O, Piiper A (2013). Differential stability of cell-free circulating microRNAs: implications for their utilization as biomarkers. PLoS One.

[13] Aleckovic M, Kang Y (2015). Regulation of cancer metastasis by cell-free miRNAs. Biochim Biophys Acta.

[14] Schwarzenbach H, Nishida N, Calin GA, Pantel K (2014). Clinical relevance of circulating cell-free microRNAs in cancer. Nat Rev Clin Oncol.

[15] Troiano G, Boldrup L, Ardito F, Gu X, Lo Muzio L, Nylander K (2016). Circulating miRNAs from blood, plasma or serum as promising clinical biomarkers in oral squamous cell carcinoma: A systematic review of current findings. Oral Oncol.

[16] Lilja H, Ulmert D, Vickers AJ (2008). Prostate-specific antigen and prostate cancer: prediction, detection and monitoring. Nat Rev Cancer.

[17] Felder M, Kapur A, Gonzalez-Bosquet J, Horibata S, Heintz J, Albrecht R, Fass L, Kaur J, Hu K, Shojaei H, Whelan RJ, Patankar MS (2014). MUC16 (CA125): tumor biomarker to cancer therapy, a work in progress. Mol Cancer.

[18] Kaskas NM, Moore-Medlin T, McClure GB, Ekshyyan O, Vanchiere JA, Nathan CA (2014). Serum biomarkers in head and neck squamous cell cancer. JAMA Otolaryngol Head Neck Surg.

[19] Lin HS, Siddiq F, Talwar HS, Chen W, Voichita C, Draghici S, Jeyapalan G, Chatterjee M, Fribley A, Yoo GH, Sethi S, Kim H, Sukari A (2014). Serum prognostic biomarkers in head and neck cancer patients. Laryngoscope.

[20] Linkov F, Lisovich A, Yurkovetsky Z, Marrangoni A, Velikokhatnaya L, Nolen B, Winans M, Bigbee W, Siegfried J, Lokshin A, Ferris RL (2007). Early detection of head and neck cancer: development of a novel screening tool using multiplexed immunobead-based biomarker profiling. Cancer Epidemiol Biomarkers Prev.

[21] Imai R, Takenaka Y, Yasui T, Nakahara S, Yamamoto Y, Hanamoto A, Takemoto N, Fukusumi T, Cho H, Yamamoto M, Inohara H (2015). Prognostic significance of serum squamous cell carcinoma antigen in patients with head and neck cancer. Acta Otolaryngol.

[22] Yamashita T, Shimada H, Tanaka S, Araki K, Tomifuji M, Mizokami D, Tanaka N, Kamide D, Miyagawa Y, Suzuki H, Tanaka Y, Shiotani A (2016). Serum midkine as a biomarker for malignancy, prognosis, and chemosensitivity in head and neck squamous cell carcinoma. Cancer Med.

[23] Fredriksson S, Gullberg M, Jarvius J, Olsson C, Pietras K, Gustafsdottir SM, Ostman A, Landegren U (2002). Protein detection using proximity-dependent DNA ligation assays. Nat Biotechnol.

[24] Gullberg M, Gustafsdottir SM, Schallmeiner E, Jarvius J, Bjarnegard M, Betsholtz C, Landegren U, Fredriksson S (2004). Cytokine detection by antibody-based proximity ligation. Proc Natl Acad Sci U S A.

[25] Assarsson E, Lundberg M, Holmquist G, Björkesten J, Thorsen SB, Ekman D, Eriksson A, Rennel Dickens E, Ohlsson S, Edfeldt G, Andersson AC, Lindstedt P, Stenvang J (2014). Homogenous 96-plex PEA immunoassay exhibiting high sensitivity, specificity, and excellent scalability. PLoS One.

[26] Boldrup L, Gu X, Coates PJ, Norberg-Spaak L, Fahraeus R, Laurell G, Wilms T, Nylander K (2017). Gene expression changes in tumor free tongue tissue adjacent to tongue squamous cell carcinoma. Oncotarget.

[27] Hsu CM, Lin PM, Wang YM, Chen ZJ, Lin SF, Yang MY (2012). Circulating miRNA is a novel marker for head and neck squamous cell carcinoma. Tumour Biol.

[28] Liu CJ, Kao SY, Tu HF, Tsai MM, Chang KW, Lin SC (2010). Increase of microRNA miR-31 level in plasma could be a potential marker of oral cancer. Oral Dis.

[29] Summerer I, Unger K, Braselmann H, Schuettrumpf L, Maihoefer C, Baumeister P, Kirchner T, Niyazi M, Sage E, Specht HM, Multhoff G, Moertl S, Belka C, Zitzelsberger H (2015). Circulating microRNAs as prognostic therapy biomarkers in head and neck cancer patients. Br J Cancer.

[30] Wong TS, Liu XB, Wong BY, Ng RW, Yuen AP, Wei WI (2008). Mature miR-184 as Potential Oncogenic microRNA of Squamous Cell Carcinoma of Tongue. Clin Cancer Res.

[31] Tiberio P, Callari M, Angeloni V, Daidone MG, Appierto V (2015). Challenges in using circulating miRNAs as cancer biomarkers.BioMed Res Int.

[32] Boldrup L, Coates PJ, Laurell G, Nylander K (2011). Differences in p63 expression in SCCHN tumours of different sub-sites within the oral cavity. Oral Oncol.

[33] Boldrup L, Coates PJ, Wahlgren M, Laurell G, Nylander K (2012). Subsite-based alterations in miR-21, miR-125b, and miR-203 in squamous cell carcinoma of the oral cavity and correlation to important target proteins. J Carcinog.

[34] Trivedi TI, Tankshali RA, Goswami JV, Shukla SN, Shah PM, Shah NG (2011). Identification of site-specific prognostic biomarkers in patients with oral squamous cell carcinoma. Neoplasma.

[35] Nylander E, Ebrahimi M, Wahlin YB, Boldrup L, Nylander K (2012). Changes in miRNA expression in sera and correlation to duration of disease in patients with multifocal mucosal lichen planus. J Oral Pathol Med.

[36] Aherne ST, Madden SF, Hughes DJ, Pardini B, Naccarati A, Levy M, Vodicka P, Neary P, Dowling P, Clynes M (2015). Circulating miRNAs miR-34a and miR-150 associated with colorectal cancer progression. BMC Cancer.

[37] Selth LA, Tilley WD, Butler LM (2012). Circulating microRNAs: macro-utility as markers of prostate cancer?. Endocr Relat Cancer.

[38] Pritchard CC, Kroh E, Wood B, Arroyo JD, Dougherty KJ, Miyaji MM, Tait JF, Tewari M (2012). Blood cell origin of circulating microRNAs: a cautionary note for cancer biomarker studies. Cancer Prev Res (Phila).

[39] Xiao C, Calado DP, Galler G, Thai TH, Patterson HC, Wang J, Rajewsky N, Bender TP, Rajewsky K (2007). MiR-150 controls B cell differentiation by targeting the transcription factor c-Myb. Cell.

[40] Lerman G, Avivi C, Mardoukh C, Barzilai A, Tessone A, Gradus B, Pavlotsky F, Barshack I, Polak-Charcon S, Orenstein A, Hornstein E, Sidi Y, Avni D (2011). MiRNA expression in psoriatic skin: reciprocal regulation of hsa-miR-99a and IGF-1R. PLoS One.

[41] Bender TP, Kremer CS, Kraus M, Buch T, Rajewsky K (2004). Critical functions for c-Myb at three checkpoints during thymocyte development. Nat Immunol.

[42] He Y, Jiang X, Chen J (2014). The role of miR-150 in normal and malignant hematopoiesis. Oncogene.

[43] Thomas MD, Kremer CS, Ravichandran KS, Rajewsky K, Bender TP (2005). c-Myb is critical for B cell development and maintenance of follicular B cells. Immunity.

[44] Nosrat CA (1998). Neurotrophic factors in the tongue: expression patterns, biological activity, relation to innervation and studies of neurotrophin knockout mice. Ann N Y Acad Sci.

[45] Louie E, Chen XF, Coomes A, Ji K, Tsirka S, Chen EI (2013). Neurotrophin-3 modulates breast cancer cells and the microenvironment to promote the growth of breast cancer brain metastasis. Oncogene.

[46] Ricci A, Greco S, Mariotta S, Felici L, Bronzetti E, Cavazzana A, Cardillo G, Amenta F, Bisetti A, Barbolini G (2001). Neurotrophins and neurotrophin receptors in human lung cancer. Am J Respir Cell Mol Biol.

[47] Ivanov SV, Panaccione A, Brown B, Guo Y, Moskaluk CA, Wick MJ, Brown JL, Ivanova AV, Issaeva N, El-Naggar AK, Yarbrough WG (2013). TrkC signaling is activated in adenoid cystic carcinoma and requires NT-3 to stimulate invasive behavior. Oncogene.

